# Effect of adding letrozole to gonadotropin on in vitro fertilization outcomes: An RCT

**DOI:** 10.18502/ijrm.v13i4.6891

**Published:** 2020-04-30

**Authors:** Maryam Eftekhar, Lida Saeed

**Affiliations:** ^1^Research and Clinical Center for Infertility, Yazd Reproductive Sciences Institute, Shahid Sadoughi University of Medical Sciences, Yazd, Iran.; ^2^Afzalipour Hospital, Kerman University of Medical Science, Kerman, Iran.

**Keywords:** Letrozole, Ovarian stimulation, Pregnancy.

## Abstract

**Background:**

Aromatase inhibitors prevent the aromatization of androgens into estrogens, which reduces the negative feedback of estrogen on the hypothalamic-pituitary axis. It is clear that increasing the secretion of follicle-stimulating hormones results in an increased follicular growth.

**Objective:**

This study aimed to evaluate the effect of adding letrozole to gonadotropin on in vitro fertilization outcomes in normal responders.

**Materials and Methods:**

In this randomized clinical trial, 100 normal responder women candidate for controlled ovarian stimulation were randomly enrolled in two groups (n = 50/each). In the case group letrozole was added to gonadotropin in the antagonist protocol. The control group received the conventional antagonist protocol. The main outcome was clinical and chemical pregnancy; and the second outcomes were the number of mature oocytes, the fertilization rate, estradiol level, and the total dose of gonadotropins.

**Results:**

Basic clinical and demographic features were comparable between the groups. Estradiol level on the day of human-chorionic-gonadotropin administration and the total gonadotropin consumption were significantly higher in the control group than the case group (p = 0.045). In addition, the number of MII oocytes was higher (but not significantl) in the case group than the control group (p = 0.09). Moreover, the endometrial thickness was significantly lower in the case group. There were no significant differences in fertilization rate and chemical and clinical pregnancy rates between the two groups.

**Conclusion:**

Although adding letrozole to gonadotropin in normal responders reduces the total dose of gonadotropin, it does not improve the pregnancy outcomes.

## 1. Introduction

Letrozole is a major aromatase inhibitor that is used to treat infertility and subfertility. The aromatase enzyme is blocked by letrozole, which inhibits the conversion of androgen into estrogen and the subsequent increase in intraovarian androgens (1). Existing studies suggest that androgens play a significant role in developing ovarian follicles (2, 3). In the process of estrogen biosynthesis, aromatase plays a rate-limiting role; suppressing this role reduces estrogen levels as well as the negative feedback caused by estrogen on the secretion of gonadotropin (1).

Stimulating the endogenous generation of gonadotropin by the feedback of negative mechanisms minimizes the necessity of exogenous gonadotropin and in vitro fertilization (IVF) cycle costs (4). Moreover, aromatase inhibitors do not diminish the estrogen receptors that exist in the endometrium and the hypothalamic-pituitary axis. In addition, apart from stimulating endogenous gonadotropin, a momentary intrafollicular-androgenic environment is created by aromatase inhibitors that could improve the follicular reaction to follicle-stimulating hormones (FSHs) through the sensitization and overexpression of FSH receptors (4-6).

Letrozole has no antiestrogenic impact on endometrium (7). In addition, in normal women who undergo in vitro fertilization (IVF), lowering the levels of follicular and serum estrogen may help to reduce the possibility of ovarian hyperstimulation syndrome (OHSS) (8). This is because a relationship has been established between the use of letrozole and a reduction in the OHSS incidence rate (9). Such reports have motivated us to suppose that the use of letrozole as a co-therapy factor in the antagonist gonadotropin-releasing hormone (GnRH) protocol in normal responders' cycles improves cycle outcomes.

Accordingly, this study aimed to compare the IVF outcomes of normal responders who have received gonadotropin both with and without the addition of letrozole during ovarian stimulation.

## 2. Materials and Methods 

In this randomized clinical trial, 100 women, aged from 18-40 yr, with normal ovarian response who were visited the Research and Clinical Center for Infertility, Yazd, Iran from August to December of 2018, and planned to undergo assisted reproduction technology (ART) were enrolled.

Normal ovarian response was considered as the antral follicular count >7, and AMH from 1.1- to 3.5 ng/ml.

Our exclusion criteria were:

• A history of endocrine abnormalities

• Intrauterine disorders (intrauterine adhesions, submucosal fibroma, and uterine polyp)

• Azoospermia of the husband

• Severe endometriosis

The participants were randomly divided into two equal-sized groups. Sealed envelopes were the basis for randomization. The case group received gonadotropin + letrozole + antagonist. The control group received antagonist + gonadotropin.

### Stimulation protocol

From the second day of the cycle, both groups were subcutaneously administered with 150 units of Cinnal-f (CinnaGen, Iran). In addition, from the second day to the trigger day, the case group was orally administered with 5 mg of letrozole (Iran Hormone Company, Iran). In the two groups, as the size of the dominant follicle reached 12-13 mm, Cetrotide (Merck, Serono, Germany) 0.25 mg/daily was subcutaneously administered. When the size of 2-3 follicles amounted to 17 mm, the last triggering stage was completed with the intramuscular administration of hCG (Pregnyl, Merck, Germany). All participants at high risk of developing OHSS (either those with a past record of OHSS, those with more than 20 follicles greater than 10 mm on the day of triggering, or those with estradiol levels greater than 4,000 pmol/l) were subcutaneously triggered with a GnRH agonist, specifically 0.2 cc of decapeptyl (Ferring Co., Germany). Endometrial thickness and serum E${}_{2}$ levels were measured on the day of hCG injection. Oocyte retrieval was performed 34-36 hr after hCG injection, and conventional IVF and/or intracytoplasmic sperm injection were conducted. Embryo grading was done by emberyologist, Grade A embryos had equal size blastomeres without fragmentation, Grade B embryos had little difference in blastomeres size, and less than 10% cytoplasmic fragments. Grade C embryos had blastomeres with Unequal sized and less than 50% fragments. Embryos were transferred using a Labotect catheter (Labotect, GmbH, Rosdorf, Germany) 48-72 hr after oocyte retrieval. The embryos of participants with a high risk of OHSS were frozen.

On the oocyte-retrieval day, progesterone suppositories (CyclogestⓇ, 400 mg) were administered vaginally two times per day for the purpose of luteal-phase support. This was maintained until ultrasonography demonstrated fetal heart activity.

Chemical and clinical pregnancy rates were the major outcomes. The secondary outcomes consisted of the fertilization rate (percentage transformation of micro injected oocytes into two pronuclei), number of mature oocytes (MII), total dose of gonadotropin, and estradiol level.

Fourteen days after embryo transfer, serum beta-hCG (β-hCG) was examined. β-hCG > 50 IU/L was regarded as a positive pregnancy test. In addition, a positive β-hCG 14 days after embryo transfer was considered as the chemical pregnancy. Transvaginal ultrasonography, which was conducted 21 days after positive β-hCG, was used to diagnose clinical pregnancy as the identification of fetal heart activities.

### Ethical consideration

This trial was approved by the ethics committee of the Research and Clinical Center for Infertility, Shahid Sadoughi University of Medical Sciences, Yazd, Iran (Code: IR.SSU.RSI.REC.1397.002). In addition, the study proposal was registered at the Iranian Registry of Clinical Trials (IRCT) (Code: IRCT2011050906420N19). The participants were informed about normal infertility treatments and IVF processes, after which they provided informed written consent.

### Statistical analysis

Statistical analysis was carried out using the Statistical Package for the Social Sciences (SPSS software), version 20.0, Chicago, Illinois. To determine the significant differences between both groups, Student's *t* test and Chi-square test were employed with the significance level set at p-value < 0.05.

## 3. Results

**Figure 1:**
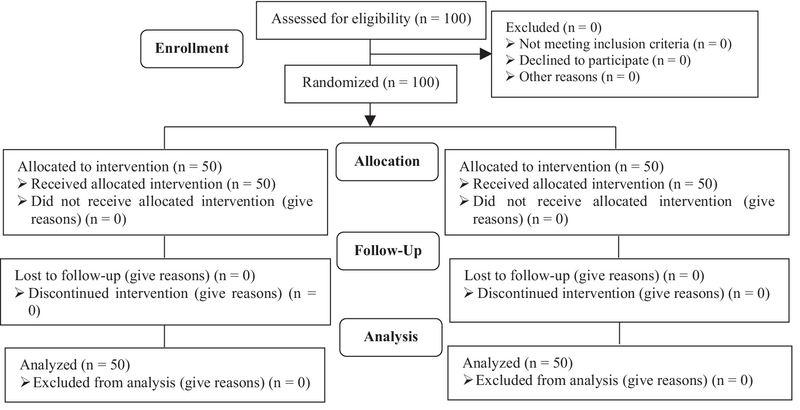
The COSORT flowchart of the study.

Totally, One hundred participants were included in this study into two groups (n = 50/each) (Figure 1). As shown in table I, two study groups were matched in the terms of baseline characteristics. On the day of hCG administration, estradiol levels and the total gonadotropin consumption were significantly higher in the control group (p = 0.045) (Table II).

The endometrial thickness (was significantly lower in the case group than the controls (p = 0.002). The number of MII oocytes was higher (but not significantly) in the case group (p = 0.09), as shown in Table II. Indeed, there were no significant differences in the fertilization rate nor the chemical and clinical pregnancy rates between the two groups (Table III). Embryos were frozen in two participants of each group because risk of OHSS.

**Table 1:** Basal characteristics of participants in two study groups (n = 50/each)


**Variable**		**Case group**	**Control group**	**p-value**
**Age (year) (mean ± SD)**		29.82 ± 4.11	29.90 ± 3.67	0.91**
**Duration of infertility (year) (mean ± SD)**		5.88 ± 3.72	6.38 ± 3.16	0.47**
**Type of infertility n (%)**				
	**Primary**	43 (86)	44 (88)	0.77 *
	**Secondary**	7 (14)	6 (12)	
**AMH (mean ± SD)**		2.65 ± 0.69	2.57 ± 0.64	0.29**
**Infertility cause n (%)**				
	**Male factor**	33 (66)	35 (70)	0.53*
	**Tubal factor**	1 (2)	1 (2)	
	**Unexplained**	16 (32)	14 (28)	
* Chi-square test, ** Student's *t* test, AMH: Anti-Mullerian hormone				

**Table 2:** Comparison of cycle characteristics in embryo transfer in two study groups (n = 50/each)


**Variable**		**Case group**	**Control group**	**p-value**
**Endometrial thickness (mm) (mean ± SD)**		7.89 ± 1.78	9.30 ± 2.53	0.002**
**No. of transferred embryos (mean ± SD)**		1.78 ± 0.50	1.67 ± 0.68	0.38**
**Embryos grade**				
	**A (%)**	41.7	34.9	0.65*
	**B (%)**	43.8	53.5	
	**C (%)**	14.6	11.6	
**OHSS n (%)**		2 (4.0)	2 (4.0)	1.00*
**Estradiol level on the day of hCG administration (pg/mL) (mean ± SD)**		311.04 ± 229.64	1350.99 ± 833.29	<0.001**
**Gonadotropin dose (IU) (mean ± SD)**		20.98 ± 5.79	23.72 ± 7.70	0.045**
**No. MII oocyte retrieved (mean ± SD)**		8.46 ± 4.73	6.96 ± 4.09	0.093**
**Freeze embryo transfer cycle n (%)**		6 (12)	5 (10)	0.75*
** Students' *t* test, * Chi-square test, OHSS: Ovarian hyperstimulation syndrome				

**Table 3:** Comparison of ART outcomes in two study groups (n = 50/each)


**Variable**	**Case group**	**Control group**	**p-value**
**Chemical pregnancy rate, n (%)**	12 (24.0)	14 (28.0)	0.65*
**Clinical pregnancy rate, n (%)**	10 (20.0)	11 (22.0)	0.8*
**Fertilization rate, (%) (mean ± SD)**	65.60 ± 24.90	62.73 ± 26.90	0.60*
** Student's *t* test * Chi-square test			

## 4. Discussion

The hypothesis of the present research indicated that the adding of letrozole to gonadotropin in the ovarian stimulation protocol in normal responder patients could enhance pregnancy outcomes and decrease gonadotropin use. Although the results showed no difference between the two groups in the clinical and chemical pregnancy rates; the gonadotropin dose, and estradiol levels decreased in the case group, significantly. Recently, many studies have shown letrozole to be advantageous in IVF cycles, particularly in patients with breast cancer and undergoing treatment to preserve fertility (9-12). Moreover, the co-administration of gonadotropin and letrozole enhances outcomes in normal responders going through IVF cycles (13-15). Lazer and co-workers compared pregnancy and IVF outcomes of poor responder patients to the treatment with high doses of gonadotropin and those treated with gonadotropin and letrozole with minimal doses. Even though no significant differences were observed in the number of oocytes retrieved and the eggs fertilized between the two protocols, a significantly higher rates of clinical pregnancy and live birth were evident in the group received minimal-dose of gonadotropin + letrozole (16). In an RCT with 94 participants, Mukherjee and colleagues examined the letrozole addition to FSH in an antagonist GnRH protocol for ovarian stimulation cycles and found no differences in the rate of clinical pregnancy (36% (15/42) vs. 33% (17/52) p-value 0.82) and the mature oocytes' number (4.6 ± 2.5 vs. 4.9 ± 2.3 (p-value 0.55)). Moreover, there were no OHSS cases in the letrozole group, but seven cases were present in the control group (17). In a similar study on normal responders, Haas J and others reported that co-treatment with letrozole enhanced the IVF treatment outcomes by increasing blastocysts and the number of mature oocytes obtained, without increasing the risk of OHSS (18). The OHSS risk rate was the same in the two groups in our study. Epidemiological research implies that, during pregnancy, any increase in the estradiol levels caused by hormone replacement therapy and oral contraceptive pills is positively correlated with an increase in the risk of thromboembolic occurrence. In the course of IVF treatment, women who develop OHSS with high levels of estradiol and hemoconcentration are more likely to be affected by thromboembolic events (19). Indeed, supraphysiological amounts of estradiol circulation can reduce the implantation rate as well as imperfect placentation, thereby causing pregnancy complications, including intrauterine growth restriction and small for gestational age, both of which can be prevented by transferring frozen embryos when the levels of circulating estrogen increase (18). The present study demonstrated that co-treatment with letrozole reduced estrogen levels, resulting in a decrease in the possibility of developing thromboembolic cases and pregnancy complications. Moreover, the quick endometrial development and the lack of antiestrogenic impact on the endometrium could be the positive effects of aromatase inhibitors in IVF cycles (20). In the RCT on 20 women, Verpoest and co-workers found out that adding letrozole to FSH in an antagonist GnRH protocol for controlled ovarian hyperstimulation did not affect the rate of ongoing pregnancies (50% vs. 20%) nor the number of the retrieved oocytes (13.8 ± 9.2 vs. 9.6 ± 7.7). Also, they reported that, on the day of administering hCG, the mean of endometrial thickness was considerably higher in the group administered with letrozole than that administered without letrozole (7). Contrastingly, in the present study, endometrial thickness was significantly lower in the group administered with letrozole. Moreover, despite the similar results in the term of cycle duration in the letrozole group, the overall the dose of gonadotropin used was significantly lower, which reduced IVF costs. In addition, the rate of clinical pregnancy and the number of transferred embryos were not different between the cycles in the presence and absence of letrozole. Indeed, the findings of the present trial verified the results of previous studies on normal responder women undergoing IVF for the diagnosis of severe male factor infertility using aromatase blockers for controlled ovarian hyperstimulation (4, 17).

## 5. Conclusion

Adding letrozole to gonadotropin in women with normal ovarian response reduces the overall gonadotropin dose; however, it does not improve pregnancy outcomes.

##  Conflict of Interest

The authors declare that they have no conflict of interests.
